# Efficacy of the integrated management model of medical care on non-attendance, satisfaction, and complications in adult patients undergoing day surgery for inguinal hernia

**DOI:** 10.12669/pjms.42.3.13309

**Published:** 2026-03

**Authors:** Qian Sun, Shuhui Zhao, Xiaoshuang Liu, Jingchao Yang, Litao Liu

**Affiliations:** 1 Qian Sun Department of General Surgery, Affiliated Hospital of Hebei University, Baoding 071000, Hebei, China; 2 Shuhui Zhao Department of Internal Medicine, Affiliated Hospital of Hebei University, Baoding 071000, Hebei, China; 3 Xiaoshuang Liu Department of General Surgery, Affiliated Hospital of Hebei University, Baoding 071000, Hebei, China; 4 Jingchao Yang Department of General Surgery, Affiliated Hospital of Hebei University, Baoding 071000, Hebei, China; 5 Litao Liu Department of General Surgery, Affiliated Hospital of Hebei University, Baoding 071000, Hebei, China

**Keywords:** Complications, Day Surgery, Integrated Model of Medical Care, Inguinal Hernia, Surgical Non-attendance, Patient Satisfaction

## Abstract

**Objective:**

To investigate the efficacy of the integrated management model of medical care on the non-attendance rate, satisfaction rate, and incidence of complications in adult patients undergoing day surgery for inguinal hernia.

**Methodology:**

This was a retrospective study. A total of one hundred patients with inguinal hernia who underwent day surgery in Affiliated Hospital of Hebei University from January 2023 to May 2025 were selected as the subjects and were divided into an Intervention group(n=50) and a control group (n=50) according to intervention methods. Conventional management was implemented in the control group, while the integrated management model of medical care was adopted in the intervention group in addition to conventional management. The surgical non-attendance rate, incidence of adverse reactions, satisfaction, incidence of complications, and quality of life(QoL) were compared between the two groups.

**Results:**

The surgical non-attendance rate and incidence of adverse events in the observation group were significantly lower than those in the control group(P<0.05). Meanwhile, patient satisfaction in the observation group was higher than the control group(χ^2^=4.762, p=0.029). Additionally, the observation group incidence of complications was lower than the control group(p<0.05). Furthermore, KPS and SF-36 QoL scores in the observation group were significantly improved as compared to those in the control group(p<0.05).

**Conclusion:**

The integrated management model of medical care in adult patients undergoing day surgery for inguinal hernia exhibits satisfactory results and may significantly reduce the surgical non-attendance rate and incidence of adverse events and complications.

## INTRODUCTION

Currently, day surgery has seen increasing application, particularly for the common clinical condition of adult inguinal hernia, where the proportion of day surgery is on the rise.^1^ In developed countries of Europe and America, approximately 80% of inguinal hernia surgeries are performed as day surgeries, and this figure in China has increased to over 50% in recent years.^2^,^3^ The day surgery model boasts significant advantages in reducing hospital stays, lowering medical costs, and improving bed turnover rates.

However, some issues related to day surgery are gradually emerging, such as a high non-attendance rate that, as shown by research, can be up to 10%-15% in day surgery for inguinal hernia,^4^ which not only leads to a serious waste of medical resources but also affects the operational efficiency of the operating room. Meanwhile, problems such as insufficient health education and inadequate postoperative guidance can arise due to the short hospital stay and limited patient-physician contact time in day surgery, resulting in lower patient satisfaction. Additionally, the lack of professional monitoring after discharge can lead to complications, impacting surgical outcomes and patient safety.^5^

Therefore, these issues hinder the promotion and application of day surgery. As a novel healthcare service model, the integrated management model of medical care emphasizes collaboration between doctors and nurses as a team to participate in the overall management of patients, providing continuous and seamless medical services. Despite multiple studies having shown^6^,^7^ significant effects of this integrated model in areas such as chronic disease management and perioperative care, its application, particularly in day surgery for inguinal hernias, lacks systematic evaluation. In this regard, this study is designed to systematically assess the application effects of the integrated management model of medical care in adult patients undergoing day surgery for inguinal hernia, thereby providing scientific evidence for clinical practice.

## METHODOLOGY

This was a retrospective study. A total of one hundred patients with inguinal hernia who underwent day surgery in the general surgery department of Affiliated Hospital of Hebei University from January 2023 to May 2025 were retrospectively selected as the subjects. Patient data were collected from the electronic medical record system of our hospital, including baseline characteristics, medical history, physical examination and laboratory examination. Afterward, patients were divided into an observation group (n=50) and a control group (n=50), with the control group following the conventional management process and the intervention group receiving the integrated management of medical care in addition to the conventional process.

### Ethical approval:

The study was approved by the Institutional Ethics Committee of Affiliated Hospital of Hebei University (No: HDFYLL-IIT-2025-044; Date: April 1, 2025), and written informed consent was obtained from all participants.

### Inclusion criteria:

- Patient clinically diagnosed with primary unilateral inguinal hernia and undergoing surgical treatment, aged ≥18 years.
- Patients who met day surgery criteria and had complete clinical data and good communication ability.
- Patients without obvious mental or neurological symptoms, with high compliance, and able to cooperate with completing the study.

### Exclusion criteria:

- Patients with severe liver or kidney dysfunction or other organic diseases.
- Patients with concurrent malignant tumors.

### Intervention methods:

Conventional Process: Doctors, nurses, and patients each completed their respective responsibilities and tasks. Specifically, outpatient doctors identified adult patients with inguinal hernia who met day surgery criteria and prescribed necessary preoperative examinations; patients completed hospital admission and preoperative preparations, arriving at the scheduled time for surgery and postoperative follow-up, including dressing changes; outpatient nurses were responsible for registering information, scheduling surgery times, and notifying patients of relevant precautions; and ward nurses completed admission and discharge procedures, perioperative care, and education on precautions.

### Integrated Management Model:

- An integrated cooperation team of medical care led by the department head and the head nurse was established by selecting senior specialized nurses from the ophthalmology department for participation. Meanwhile, a process optimization group consisting of specialized nurses and ward doctors from day surgery and ward departments was formed, including the surgical doctors in a WeChat group for integrated day ward management;
- The communication system, performance evaluation system, job management system, training system, etc. were developed for the integrated management of medical care;
- Tasks of the process optimization group were clearly defined, with members collectively reviewing the original process and assessing existing problems and causes, and a patient-centered process optimization plan that reflected simplicity, safety, and efficiency was designed;

Relevant measures for process optimization were collaboratively implemented among doctors, nurses, and patients, along with an observation of evaluation indicators. This manuscript conformed the Enhancing the Quality and Transparency Of health Research (EQUATOR) network guidelines.

### Observational indicators:

*Non-attendance rate for day surgery:* It refers to the ratio of hospitalized patients who, for various reasons, fail to arrive at the hospital on the scheduled date after registering for surgery to the total number of patients;

### Incidence of adverse events:

This indicates the ratio of occurrences of adverse events to the total number of surgeries performed. Adverse events include any medical or nursing anomalies intercepted or occurring at various stages, such as incorrect surgical site marking, missing medical documents, and identity verification errors;

### Comparison of patient satisfaction:

Patient satisfaction was analyzed using a simplified questionnaire pre- and post-intervention, including items such as very satisfied, somewhat satisfied, satisfied, uncertain, and dissatisfied. The overall satisfaction was calculated as follows: Overall Satisfaction = (Very Satisfied + Somewhat Satisfied + Satisfied) / Total Cases×100%;

### Comparison of postoperative complications:

This included evaluating the occurrence of complications in both groups, such as infection, bleeding, and poor wound healing;

### Postoperative recovery:

The postoperative recovery for both groups were recorded, including the time to ambulation and the duration of postoperative hospitalization;

Comparison of the improvement in functional status and quality of life pre- and post-surgery for both groups using KPS scores (Karnofsky Performance Status scale) and QoL scores (SF-36 Quality of Life scale).

### Statistical analysis:

SPSS 22.0 was used for the statistical analysis of data. According to the data of each indicator in the pre-survey, the sample size is estimated by 95% confidence interval, and the largest one is the sample size of the study. The sample size required for each group was ≥50 cases on the basis of Fisher exact probability. Measurement data were expressed as Mean±SD (*x̅*±*s*), and independent sample t-tests were utilized for pre- and post-treatment comparisons. Count data were expressed as n (%), and inter-group comparisons were conducted using the χ² test. P < 0.05 was considered significantly different.

## RESULTS

The observation group: 36 males and 14 females, aged 26-74 years (average: 58.50±7.33 years). The control group: 34 males and 16 females, aged 24-75 years (average: 57.70±7.04 years). No significant differences were observed in gender and age between the two groups (P>0.05), indicating comparability.

The surgical non-attendance rate and incidence of adverse events in the observation group were 6.00% and 4.00%, respectively, both remarkably lower than the 20.00% and 16.00% in the control group, with significant differences (P<0.05) (Table-I).

**Table-I:** Comparison of surgical non-attendance and adverse events between the two groups [n(%)].

Group	Surgical Non-attendance	Adverse Events
Observation(n=50)	3(6.00)	2(4.00)
Control(n=50)	10(20.00)	8(16.00)
*χ* ^2^	4.332	4.000
*P*	0.037	0.046

The observation group reported a patient satisfaction of 92%, notably higher than 76.00% in the control group, and the differences were statistically significant (p<0.05) (Table-II).

**Table-II:** Comparison of patient satisfaction between the two groups[n(%)].

Group	Very satisfied	Somewhat satisfied	Satisfied	Uncertain	Dissatisfied	Overall satisfaction
Observation(n=50)	30(60.00)	10(20.00)	6(12.00)	3(6.00)	1(2.00)	46(92.00)
Control(n=50)	22(44.00)	9(18.00)	7(14.00)	7(14.00)	5(10.00)	38(76.00)
*χ* ^2^						4.762
*P*						0.029

Postoperative complications in the two groups were mainly incision infection, poor incision healing, and postoperative bleeding. The incidence of complications in the observation group was 7.50%, lower than 25.00% in the control group, with statistically significant differences (p<0.05) (Table-III). The time to first ambulation and time to return to normal life in the observation group were substantially shorter than those in the control group, with statistically significant differences (p<0.05). (Table-IV).

**Table-III:** Comparison of incidences of complications between the two groups (*χ̅*±*S*).

Group	Incision Infection	Postoperative Bleeding	Nausea/ Vomiting	Poor Incision Healing	Incidence
Observation	1(2.00)	1(2.00)	0(0.00)	1(2.00)	3(6.00)
Control	3(6.00)	2(4.00)	4(8.00)	1(2.00)	10(20.00)
*χ* ^2^					4.332
*p*					0.037

**Table-IV:** Comparison of postoperative recovery between the two groups (*χ̅*±*S*).

Group	Time to Ambulation(h)	Time to Return to Normal Life(d)
Observation	19.40±5.14	3.72±0.45
Control	26.24±5.57	4.30±0.86
*t*	6.380	4.206
*P*	0.000	0.000

No significant differences were observed in preoperative KPS and SF-36 QoL scores between the two groups (p>0.05). However, these indicators in the observation group were remarkably improved compared with the control group postoperatively, with statistically significant differences (p<0.05) (Table-V).

**Table-V:** Comparison of pre- and post-surgery QoL scores between the two groups (*χ̅*±*S*).

Indicator		Observation Group	Control Group	t	p
KPS Score	Pre-surgery	64.56±9.83	64.44±8.38	0.066	0.948
	Post-surgery	73.40±10.90	67.66±9.58	2.797	0.006
SF-36 Score	Pre-surgery	46.12±6.84	46.16±7.08	0.029	0.977
	Post-surgery	62.42±8.30	53.18±7.54	5.828	0.000

## DISCUSSION

The findings of this study indicate that the integrated management model of medical care significantly reduced the non-attendance rate and incidence of adverse events in adult patients undergoing day surgery for inguinal hernia. Research has shown^8^,^9^ that providing patients with adequate education preoperatively can reduce anxiety and improve compliance. The integrated management model of medical care consists of a multidisciplinary collaborative team, including doctors, nurses, and anesthetists, who collectively participate in patient management and enhance patients’ understanding of the surgical process, anesthesia methods, and postoperative recovery through systematic communication, thereby reducing surgical non-attendance due to fear or misunderstanding. Literature indicates^10^,^11^ that non-attendance among patients for day surgery is often related to forgetfulness or scheduling conflicts. In this regard, the integrated management model of medical care strengthens preoperative appointment management through information technologies (e.g., SMS reminders, follow-ups via phone), which reduces the occurrence of surgical non-attendance and the incidence of adverse events. Moreover, the integrated management team of medical care can provide targeted interventions for high-risk patients, such as the elderly or those with lower education levels, thereby further decreasing surgical non-attendance.^12^ Under the conventional model, the main subjects taking responsibility for preoperative, intraoperative, and postoperative operations are decentralized, easily leading to information disconnection. By contrast, integrated management involves a fixed team responsible throughout the process, enhancing patient safety and trust.^13^ It has been shown^14^ that explaining surgical steps with visual tools (e.g., animations and manuals) during preoperative visits and developing rehabilitation plans tailored to individual patient needs can improve patient satisfaction. In 2018, patient satisfaction was recognized by the World Health Organization as a measure of safety and quality in the healthcare industry, with a global consensus on its importance.^15^,^16^

This study demonstrates a significantly higher patient satisfaction in the observation group than in the control group, suggesting that the patient-centered, goal-oriented, and clearly defined integrated management model breaks down the previously parallel pathways of doctor-patient and nurse-patient interactions, establishing a triadic working relationship among doctors, nurses, and patients. Therefore, this approach better meets the informational needs of patients and enhances the quality of hospital services, inevitably increasing patient satisfaction with the work of healthcare personnel.^17^ Meanwhile, the findings of this study reveal that the incidence of postoperative complications in the observation group was significantly lower than that in the control group, and the time to first ambulation and the time to return to normal life were both remarkably shorter than those in the control group. It is believed that the reason for this is that the integrated team of medical care established a standardized perioperative pathway, including the use of prophylactic antibiotics and guidance for early ambulation. Through comprehensive risk assessment of patients and targeting potential complications, the team reduced the risk of complications while ensuring that patients received comprehensive and professional medical care during the perioperative period, thereby facilitating quick recovery.

In this study, the KPS and SF-36 QoL scores of patients in the observation group substantially improved compared with those in the control group, suggesting that the integrated management model of medical care is beneficial for reducing postoperative complications and improving the quality of life of patients. The reason for this is that conventional care focuses on the treatment and nursing of diseases, often neglecting the overall quality of life of patients. By contrast, the integrated management model of medical care addresses medical care work from various aspects of the perioperative period and strengthens health education while providing comprehensive and personalized services, which not only focuses on the physiological health of patients but also pays attention to other aspects such as their psychological health and social functioning, effectively enhancing their quality of life. This is consistent with findings reported by multiple studies.^18^-^20^

### Limitations:

It is a single-center study with a relatively small sample size and a short follow-up period, and no assessment was conducted on changes in the workload of healthcare personnel. Therefore, it is necessary to expand the sample size, increase the duration of follow-up, and include more outcome indicators in future clinical work, so as to provide more comprehensive evidence support for clinical decision-making.

## CONCLUSIONS

The integrated management model of medical care may significantly reduce the non-attendance rate and incidence of adverse events in adult patients undergoing day surgery for inguinal hernia, improve patient satisfaction, and reduce the incidence of postoperative complications, ultimately enhancing the quality of life of patients.

### Authors’ Contributions:

**QS** and **SZ:** Performed the studies, data collection, and drafted the manuscript, and are responsible and accountable for the accuracy or integrity of the work.

**XL** and **JY:** Study design, Statistical analysis and critical review.

**LL:** Designed, data analysis and write up. All authors read and approved the final manuscript.
